# The Forward Model: A Unifying Theory for the Role of the Cerebellum in Motor Control and Sense of Agency

**DOI:** 10.3389/fnsys.2021.644059

**Published:** 2021-04-15

**Authors:** Quentin Welniarz, Yulia Worbe, Cecile Gallea

**Affiliations:** ^1^INSERM U-1127, CNRS UMR 7225, Institut du Cerveau, Faculté de Médecine, Sorbonne Université, La Pitié Salpêtrière Hospital, Paris, France; ^2^Movement Investigation and Therapeutics Team, ICM, Paris, France; ^3^Department of Neurophysiology, Saint-Antoine Hospital, Assistance Publique–Hôpitaux de Paris, Paris, France

**Keywords:** prediction error, sensory mismatch, neuroimaging, motor control, movement disorders

## Abstract

For more than two decades, there has been converging evidence for an essential role of the cerebellum in non-motor functions. The cerebellum is not only important in learning and sensorimotor processes, some growing evidences show its implication in conditional learning and reward, which allows building our expectations about behavioral outcomes. More recent work has demonstrated that the cerebellum is also required for the sense of agency, a cognitive process that allows recognizing an action as our own, suggesting that the cerebellum might serve as an interface between sensorimotor function and cognition. A unifying model that would explain the role of the cerebellum across these processes has not been fully established. Nonetheless, an important heritage was given by the field of motor control: the forward model theory. This theory stipulates that movements are controlled based on the constant interactions between our organism and its environment through feedforward and feedback loops. Feedforward loops predict what is going to happen, while feedback loops confront the prediction with what happened so that we can react accordingly. From an anatomical point of view, the cerebellum is at an ideal location at the interface between the motor and sensory systems, as it is connected to cerebral, striatal, and spinal entities *via* parallel loops, so that it can link sensory and motor systems with cognitive processes. Recent findings showing that the cerebellum participates in building the sense of agency as a predictive and comparator system will be reviewed together with past work on motor control within the context of the forward model theory.

## Introduction

Over the past decades, there has been accumulating evidence suggesting that the role of the cerebellum goes far beyond motor control and involves a variety of cognitive tasks (Strick et al., 2009). The unique architecture of the cerebellum could explain its involvement in such a diverse range of functions. The connectivity between the cerebellum and the cerebral cortex is organized in parallel loops: different regions of the cerebellum receive inputs from a large set of cerebral regions (not only from motor regions, but also from associative areas) through the pontine nuclei (PN), and in return, the deep cerebellar nuclei send projections back to the same cerebral regions through the thalamus, thus forming a Cerebro-Ponto-cerebello-dentato-thalamocortical pathway (Ito, 2006; Sokolov et al., 2017; Diedrichsen et al., 2019; Cabaraux et al., 2020; Tanaka et al., 2020). Contrary to the neocortex, the local circuitry of the cerebellum is highly uniform across its different regions, suggesting that the diversity of cerebellar functions could rely on a single cerebellar computation that would be embedded in parallel cerebro-cerebellar loops (Diedrichsen et al., 2019). Among the proposals for this single cerebellar algorithm, the “forward model” is of particular interest (Sokolov et al., 2017; Diedrichsen et al., 2019; Tanaka et al., 2020).

**Figure 1:**
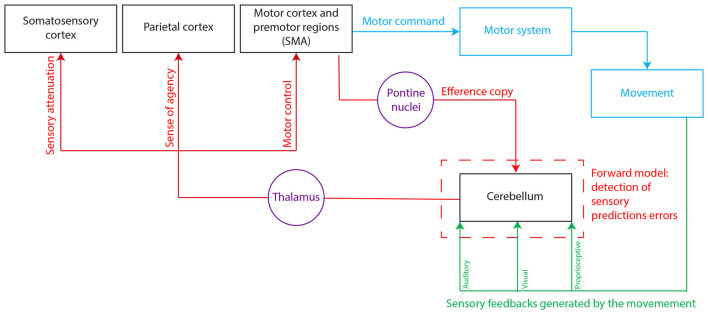
Hypothetical model for the cerebro-cerebellar loops involved in the forward model. The cerebellum is thought to integrate the efference copy—a copy of the motor command that originates from the motor and premotor areas and represents the intentional content of the action—and the actual sensory feedback generated by the movement. The existence of a discrepancy between the predicted and actual motor outcome (or sensory prediction error) would be detected at the level of the cerebellum. This signal error would then be sent to different cortical areas to serve different functions: motor control, sensory attenuation, and sense of agency. SMA, supplementary motor area.

The forward model is a computational model of voluntary motor control that emphasizes the critical role of the comparison between the intentional content of our actions and their outcomes. It was first proposed as a model to control arms and eye-movement systems (Sperry, 1950), but has now reached recognition to apply to a larger repertoire of human actions (Imamizu and Kawato, 2012). The forward model evaluates the input-output function of body segments involved in the movements and relies on two core functions: prediction and error processing (Sokolov et al., 2017). According to this model, a copy of the motor command, the “efference copy” representing the motor intention, is generated during the preparation of voluntary movements to predict the sensory consequence of the forthcoming action. This efference copy is sent to brain areas named “comparators” that monitor the congruence between the efference copy and the actual sensory feedback generated by the movement (Miall et al., 1993; Wolpert et al., 1995; Blakemore and Sirigu, 2003; Haggard and Whitford, 2004; Haggard, 2008; Jeannerod, 2009; Waszak et al., 2012; Dogge et al., 2019; Seghezzi et al., 2019; Tanaka et al., 2020). In particular, this model predicts an increased activation in the *comparator* areas in case of a sensory prediction error, i.e., when there is a mismatch between the motor command and the sensory feedback. Mismatch detection and the forward model are involved in different aspects of motor execution: in motor control (for rapid online movement adaptation, for sensory attenuation), in motor learning (sensorimotor adaptation), but also for the sense of agency (Miall et al., 1993; Wolpert et al., 1998; Desmurget and Grafton, 2000; Blakemore and Sirigu, 2003; Jeannerod, 2009; Haggard, 2017; Dogge et al., 2019; Seghezzi et al., 2019). The sense of agency can be defined as the “experience of controlling our own actions, and through them, events in the outside world” (Haggard, 2017). The sense of agency is an important cognitive process underlying action execution, as it links motor control and the feeling of being the author of our own actions. The forward model plays a crucial role in two mechanisms that occur during action execution: it ensures proper motor control and contributes to the sense of agency (Haggard, 2017). It is thus possible that the brain regions underlying motor control through a forward model could also contribute to the sense of agency. Among these areas, the cerebellum is of particular interest and could be a *comparator* considering its unique architecture and connectivity (Miall et al., 1993; Wolpert et al., 1998; Desmurget and Grafton, 2000; Blakemore and Sirigu, 2003; Ito, 2006, 2008; Jeannerod, 2009; Tanaka et al., 2020; Figure 1).

In the first part, we will define self-agency and the underlying brain network to show how motor control and the forward model are related to this concept. In the second part, we will describe the functional neuroanatomy of the cerebellum and emphasize how its singular organization is relevant regarding the forward model theory and self-agency. In a third part, we will review the functional evidence supporting the role of the cerebellum as a *predictor* and a *comparator*, in particular the fact that this region is sensitive to a sensory prediction error, i.e., to a mismatch between the intentional content of the action and its outcome, that are key processes for self-agency. We will also review the role of the cerebellum in the different functions related to motor control and learning that have been associated with the forward model and that can influence the prediction system important to self-agency. In a fourth part, we will review recent evidence linking the cerebellum to the sense of agency and we will discuss how the cerebellum could be a node of the self-agency network as a comparator and mismatch detection system.

## The Sense of Agency and The Forward Model

### Framework

In the present work, we will use an operational definition of self-agency, “the experience of controlling our own actions, and through them, events in the outside world” (Haggard, 2017). Because it is a subjective feeling, the sense of agency has been mainly studied in humans, although some pieces of evidence are suggesting that non-human primates also have a sense of agency (Kaneko and Tomonaga, 2011; Couchman, 2012). Fitting with this conception introduced by Patrick Haggard, we will consider the sense of agency as the experience that occurs before, during, and after the actual movements. In daily life, we do not often question whether we are the agent of our actions. It rather comes as an element of surprise, when unexpectedly, we detect an incongruence between our intention and the action outcome. This occurs for instance when you are fully focused on a skilled motor task (writing between two tiny lines, playing “Tretris” on your smartphone), and a neighboring person collides with you and disturbs your neat movement. This induces a discrepancy between what you planned on doing and the actual results of the action, and the feeling that you are not responsible for the action results. This example underlines that certain conditions are necessary for the sense of agency to occur. First, it requires a voluntary movement, which we refer to as an action that is purposefully initiated by the subject, by opposition to involuntary movements such as reflexes or movements caused by external devices (Haggard, 2017). Second, the expected and actual results of the action need to be compared, so that sense of agency is related to the action goals. Overall, the feeling of self-agency depends on all the steps of voluntary action and links the intention to the movement outcome. Volition and agency are two concepts that are linked (Hallett, 2010; Haggard, 2017): the first is the sense of willing a movement, the second is the sense that the willed movement has occurred. An involuntary movement could disrupt or decrease the sense of agency by inducing a discrepancy between the predictions and the actual results of the action. The neuroanatomical bases of what makes the difference between involuntary and voluntary movements are not clear cut (Hallett, 2010), especially in pathologies when both occur at the same time. The interpretation of voluntariness seems to arise from a feed-forward neural signal, a corollary discharge. The comparator that processes predictions signals from the corollary discharge and the actual movement results would help to then give rise to the sense of agency.

The sense of agency is usually divided into two distinct steps. The first one is referring to the low-level, non-conceptual, implicit feeling of control over an action, without a relationship to any conscious thought, and is known as implicit agency, feeling of agency, or instrumental agency (Synofzik et al., 2008b; Haggard, 2017). The second is referring to the explicit judgment of being the source of the action-outcome, also known as “judgment of agency” or explicit agency (Synofzik et al., 2008b). The explicit judgment of agency requires one to attribute sensory events to one’s intentional action, and is influenced by cognitive biases (such as positive outcomes); on the contrary, implicit measures capture an instinctive feeling without the need to explicitly think about agency or control, and thus are less prone to cognitive biases (Haggard, 2017). The feeling of agency and judgment of agency are two distinct processes that both contribute to the sense of agency.

Asking a subject whether he thinks he was the author of a given action is an easy way to assess the judgment of agency. By contrast, the measure of the implicit feeling of agency requires a specific experimental set-up. One implicit measure of self-agency is intentional binding. It focuses on time perception, which is influenced by the processes involved in voluntary movement. Intentional binding is the instinctive compression of the perceived time interval between an action and its outcome. In other words, the perceived time of voluntary action is shifted towards the subsequent outcomes (a sensory event following the action, such as a tone), and the perceived time of the outcomes themselves (in this example, the tone) are perceived shifted towards the voluntary actions that caused them (Haggard, 2017). As a result, intentional binding refers to the degree of control that we have over our actions (Beck et al., 2017). Sensory attenuation is an important factor influencing self-agency (Blakemore et al., 1998; Beck et al., 2017). Sensory attenuation refers to the fact that the sensory consequences of our actions are perceived differently from identical sensory input when it is externally generated: for instance, a self-produced tactile stimulus is perceived as less ticklish than the same stimulus generated externally (Blakemore et al., 1998). According to the forward model theory, sensory attenuation results from a comparison between the anticipation of movement outcome (through the efferent copy) and the actual consequence of the movement. This comparison allows us to distinguish sensory events produced by our own actions from those produced by external events, which is an important process to establish the sense of agency (Haggard, 2017). It was suggested that sensory attenuation (when stimuli are self-administrated compared to externally administrated) and outcome binding may track a common underlying process related to the sense of agency. Alternatively, perceived stimulus intensity and intentional binding could be linked by a domain-general mechanism such as multisensory cue integration (Beck et al., 2017). Last, one way of evaluating the implicit feeling of agency is to manipulate the sensory feedback (by the introduction of a delay between an action and its outcome or by the distortion of visual feedback for instance) in order to induce a mismatch with the subject actual movement. This results in a “non-agency” or “disrupted-agency” feeling, as opposed to the “positive agency” (when the predicted and actual movement outcome match).

### Networks Involved in the Sense of Agency

Over the last two decades, the use of neuroimaging techniques has led to the identification of the cerebral networks involved in the sense of agency. As stressed in the previous paragraph, the different measures of the sense of agency (explicit judgment of the agency, “non-agency” and intentional binding) have been associated with different brain regions, that have been recently reviewed in meta-analyzes (Sperduti et al., 2011; Seghezzi et al., 2019; Zito et al., 2020). The judgment of agency has been linked with activation in the anterior prefrontal cortex, the orbitofrontal cortex, the fronto-median cortex (Spengler et al., 2009; Miele et al., 2011; Zito et al., 2020), indicating that this process requires high-order, conceptual mechanisms. The network that is activated when the agency is disrupted consistently involves the temporo-parietal junction (TPJ) or inferior parietal lobule (IPL), the dorsomedial prefrontal cortex, the precuneus, the pre-supplementary motor area (preSMA), the superior and middle temporal gyrus, the angular gyrus (Sperduti et al., 2011; Seghezzi et al., 2019; Zito et al., 2020). By contrast, the “positive” self-agency has been associated with the insula, the primary somatosensory cortex, the premotor cortex, the SMA, the calcarine sulcus, the cerebellum (Sperduti et al., 2011; Seghezzi et al., 2019). Thus, it appears that the neurological substrate underlying the “two steps” of agency—the feeling of agency and the judgment of agency—is organized according to a rostrocaudal gradient in the human brain. While the feeling of agency, which has mainly been explored through the “non-agency” paradigm, seems to primarily rely on the posterior parietal cortex, the higher-order, conceptual step of the judgment of agency is implemented in prefrontal areas.

### What Is the Link Between the Sense of Agency and the Forward Model Theory?

The “comparator model” of agency suggests that the low-order, sensorimotor process underlying the implicit feeling of agency relies on a forward model (Synofzik et al., 2008b; Jeannerod, 2009; Haggard, 2017). This model relies on the prediction of the sensory consequences of actions based on the original motor commands. The comparison between the prediction of the sensory consequences of the action to the actual movement feedback is used to produce a “prediction error”. In the case of “positive” self-agency, the actual feedback fits exactly to the prediction, and the result of the comparison is zero when the event is caused by one’s action (the internal predictive model is correct in that case); otherwise, in case of a mismatch between the anticipated and the actual outcome of the movement, the result is a negative prediction error (Haggard, 2017). However, the comparator model is not sufficient to explain the higher-order level of the judgment of agency: the prediction error would then be transmitted to higher-order associative areas in the prefrontal cortex, where it would be integrated along with contextual knowledge and belief reasoning to give rise to the judgment of agency (Synofzik et al., 2008b).

## Functional Neuroanatomy of The Cerebellum Relevant to Forward The Model Theory

**Figure 2:**
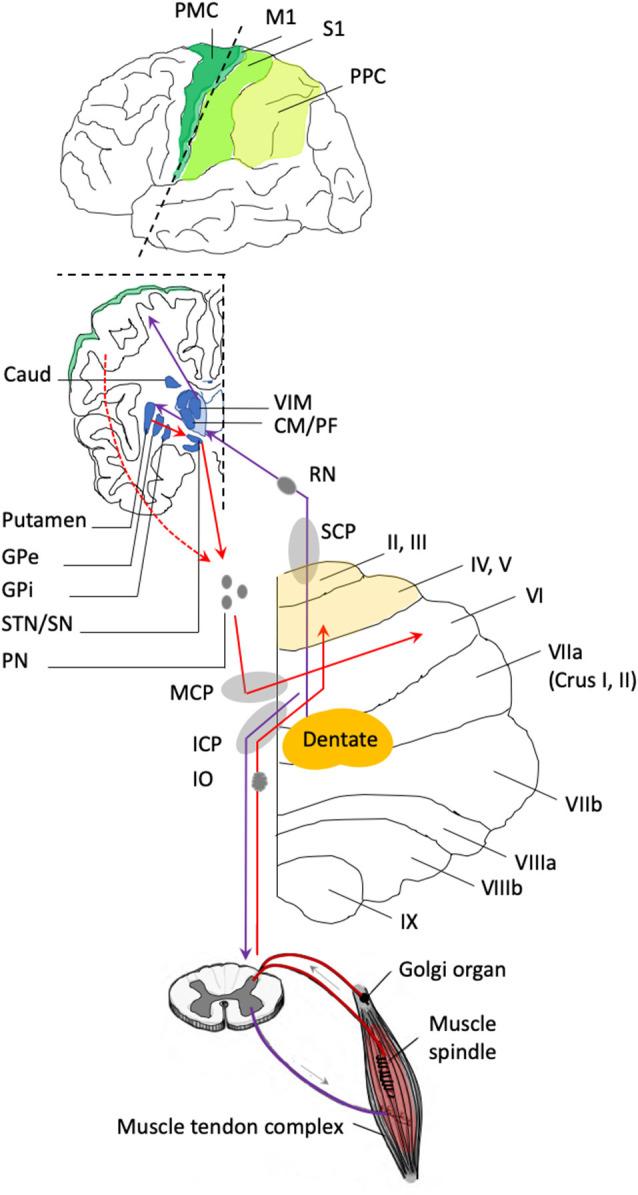
Anatomical connections of the cerebellum relevant to the forward model. The anterior lobe of the cerebellum is represented in light yellow and includes the lobules labeled from I to V. The posterior lobe of the cerebellum includes the lobules labeled from VI to IX. The functional connectivity of the cerebellum is organized in a series of loops, where the cerebellum receives inputs from the cerebral cortex, the striatum, and the spinal cord, and in return, the deep cerebellar nuclei send projections back to these same regions. **Incoming pathways** to the cerebellum are represented in red. The middle cerebellar peduncle (MCP) contains the fibers that project from the cerebral cortex and the striatum (caudate (caud) and putamen) to the posterior lobe of the cerebellum through a relay in the pontine nuclei (PN). The cerebellum receives input from a large number of cortical areas, including regions associated with motor preparation and execution (represented in green; PMC, premotor cortex; M1, primary motor cortex; S1, primary somatosensory cortex; PPC, posterior parietal cortex). The cerebellum also receives inputs from the basal ganglia (represented in blue). The subthalamic nucleus (STN) is an additional relay between the striatal output [globus pallidum pars interna (GPi) and pars externa (GPe)] and the pontine nuclei. Also, the anterior cerebellum receives sensory (proprioceptive) inputs from the spinal cord and the inferior olive that pass through the inferior cerebellar peduncles (ICP). **Outgoing pathways** from the cerebellum are represented in purple. The superior cerebellar peduncle (SCP) contains the fibers that project to the red nucleus (RN) and to the thalamic nuclei that relay the information to the cerebral cortex and the striatum. The thalamic nuclei include the ventral intermediate nucleus (VIM), which is the relay between the cerebellum and cortical brain areas; the centro-medial (CM) nucleus and the parafascicular (PF) nucleus, which are the relay between the cerebellum and the striatum. The inferior cerebellar peduncle (ICP) contains fibers that project from the cerebellum to the spinal cord.

The cerebellum is a complex structure that is connected with the entire central nervous system (Stoodley and Schmahmann, 2018). The connectivity of the cerebellum is organized in a series of parallel loops with the cerebral cortex, the striatum, and the spinal cord, which makes the cerebellum a key sensorimotor interface: each region of the cerebellum receives inputs from a specific region of the central nervous system, and sends back projections to these same regions (Sokolov et al., 2017; Diedrichsen et al., 2019; Cabaraux et al., 2020; Tanaka et al., 2020; Figure 2). *Via* the **afferent** connections, it receives information from the cerebral cortex and processes sensory feedback from the peripheral system (muscles, joint position, auditory, visual, vestibular, and proprioceptive information; Baumann et al., 2015). The middle cerebellar peduncle (MCP) conveys inputs from a large set of brain regions (not only motor areas but also associative areas) and deep brain nuclei [striatum, subthalamic nucleus (STN)] that are relayed by the pontine nuclei (Bostan and Strick, 2010; Milardi et al., 2016; Cacciola et al., 2017; Bostan et al., 2018; Diedrichsen et al., 2019). The inferior cerebellar peduncle contains the afferent information from the spinal cord (spino-cerebellar tract and inputs from the inferior olivary nucleus), including the muscle spindles, joint receptors, and Golgi tendon organs (Cullen, 2011). Thus, the cerebellum is ideally located to integrate both the motor command (or efference copy), which originates from the motor cortex and the sensory feedback generated by the movement. This singular position makes it a good candidate to be a “comparator” in the framework of the forward model, that could detect a mismatch between the motor command and the sensory feedback (Miall et al., 1993; Ito, 2006, 2008; Sokolov et al., 2017; Diedrichsen et al., 2019; Tanaka et al., 2020). This error signal would then be sent back to the cortical brain areas and spinal cord *via* the **efferent** connections, to adapt the motor output to the constant changes of our environment. The cerebellar outputs are conveyed by the deep cerebellar nuclei: the superior cerebellar peduncle (SCP) contains all the efferent white matter fibers toward the cerebral cortex that are relayed by the red nucleus (RN) and thalamic nuclei, while the inferior cerebellar peduncle contains the outputs to the spinal cord that target motoneurons and body muscles *via* the vestibular nuclei and reticular formation (Cullen, 2011).

This connectivity pattern is remarkably conserved across the different cerebellar regions. From a macroscopic point of view, the cerebellum is divided into two lobes (anterior and posterior) that are separated by the primary fissure. Each of them is further parcellated in different lobules (labeled from I to IX) that are associated with various functions, ranging from sensorimotor, cognitive, and emotional processes (Schmahmann et al., 2019). The microstructural organization of each cerebellar lobule is identical, consisting of cerebellar modules (Ito, 2006, 2008). The Purkinje cells receive excitatory inputs from the axons of granule cells (the parallel fibers) that relay the mossy fibers. In humans, there is a striking expansion of the information, as approximately 250 million mossy fibers contact 50 billion granule cells, followed by compression through 15 million Purkinje cells (Sanger et al., 2020). The Purkinje cells also receive excitatory inputs from the climbing fibers that originate in the inferior olivary. Also, the mossy fibers and climbing fibers provide excitatory inputs to the deep cerebellar nuclei. The Purkinje cells are the sole output of the cerebellar cortex and provide an inhibitory signal to their target neurons in the deep cerebellar nuclei. This specific architecture of the cerebellar modules allows a single Purkinje cell to integrate both the efference copy from the motor cortex and sensory feedback from the periphery, thus forming an adequate anatomical and functional substrate for a forward model (Cabaraux et al., 2020). In particular, learning in the cerebellum is driven by error signals (Doya, 2000; Hikosaka et al., 2002). These signals would be conveyed by climbing fibers originating from the inferior olive that encode sensorimotor information (Kitazawa et al., 1998), then integrated into Purkinje cells (Wang et al., 2000), and transmitted to the cerebral cortex *via* dentate-thalamic relays. Conceptually, motor commands originating from the cerebral cortex are optimized in terms of their sensorimotor accuracy, by going through the cerebellar loop circuits, this process being critical for motor skill learning.

Overall, the cellular microcircuitry and macroscopic connectivity pattern of the cerebellum provide the critical neural substrates of its putative role in the forward model, and thus for the feeling of agency.

## The Cerebellum as A Predictor and Comparator

A forward model relies on two core processes: prediction and detection/processing of prediction errors (Tanaka et al., 2020). In the following section, we will review the evidence supporting the role of the cerebellum in prediction and in the detection of sensory prediction errors that are key processes involved in determining self-agency. Last, we will report evidence showing that the cerebellum is also involved in anticipating self-generated movements, resulting in sensory attenuation that is linked to self-agency.

### Anticipatory Responses

Cerebellar activity is observed during the period preceding movement onset. For instance, a bilateral cerebellar activity was shown during the preparatory period of sequential finger movements (Cui et al., 2000). Pre-movement potentials were recorded in the VIM nucleus (a relay of the cerebello-cortical pathway) targeted during deep brain stimulation surgery in tremor patients (Paradiso et al., 2004; Purzner et al., 2007). When confronted with predictable perturbation, cerebellar activity in monkeys is modulated during the period preceding adaptive hand responses (Dugas and Smith, 1992; Monzée and Smith, 2004). Studies of patients with cerebellar lesions suggest that the cerebellum is involved in updating the prediction of the sensory consequences of movements to inform the perception of self-actions (Synofzik et al., 2008a; Roth et al., 2013). When dropping a ball with one hand and catching it with the other hand, the EMG pattern of the receiving hand in healthy subjects shows an anticipation process. This is not the case in cerebellar patients, suggesting that the cerebellum is involved in predicting the consequences of self-generated movements (Nowak et al., 2007). Specifically, the cerebellum might contribute to the action preparatory activity by facilitating the transitions between cortical activity states, thus contributing to adaptable and timely appropriate response (Li and Mrsic-Flogel, 2020).

Electrophysiological studies in non-human primates explored the relationship between the activity of Purkinje cells and different behavioral parameters (movement kinematics and dynamics) to precise the role of the cerebellum as an internal model (Tanaka et al., 2020). The underlying assumption was the following: a correlation between the Purkinje cells firing rate and the movement kinematics (the trajectory of the hand for instance) would be in favor of the forward model. By contrast, a correlation of the Purkinje cells activity and the movement dynamic (muscle activity) would suggest that the cerebellum functions as an inverse model that transforms the desired goal into a motor command. These experiences raised contradictory results, showing that Purkinje cells firing rates correlated either with the movement kinematics or with the movement dynamics (Pasalar et al., 2006; Yamamoto et al., 2007; Tanaka et al., 2020). A recent study clarified these controversial results by showing that the dentate nuclei cells firing rates could predict the future inputs to the cerebellum, strongly supporting the forward model (Tanaka et al., 2019). At the cellular level, this model postulates that the sensory feedback is conveyed by mossy fibers, that the prediction is computed by the Purkinje cells, and that the comparison between the sensory feedback and the prediction is operated at the level of the deep cerebellar nuclei (Tanaka et al., 2020).

### Detection of Sensory Prediction Errors

The cerebellum has been repeatedly associated with mismatch detection, a process that is essential for the feeling of agency. First, activity in the cerebellum is increased during motor errors (Diedrichsen et al., 2005; Schlerf et al., 2012). Second, activity in the cerebellum is modulated when the sensory feedback (visual, auditory, or tactile) generated by the subject’s movement is manipulated. This manipulation induces a discrepancy between the initial motor intention and the prediction of the movement outcome on the one hand, and the actual sensory consequences of the movement on the other hand. In particular, when introducing a variable and unexpected delay between the subject’s movement and its sensory consequences (tactile or visual), the activity in the cerebellum positively correlated with this delay (Blakemore et al., 2001; van Kemenade et al., 2019). In another study, the subjects were instructed to make hand movements while receiving real-time visual feedback of a simulated hand. The simulated hand was either visually synchronous with the subject’s movements, or not with a varying degree of mismatch. This procedure induced a loss of control of the hand based on the manipulated visual feedback and was associated with increased activation in the cerebellum among other regions (Nahab et al., 2011).

It thus appears that the cerebellum computes sensory prediction errors (sPE), which relate to the error between the sensory outcome and its prediction (Wolpert and Flanagan, 2001). Two mechanisms could be at play: the cerebellum may process errors as unexpected sensory events or may signal both the occurrence of unexpected stimuli and the omission of expected stimuli (Schlerf et al., 2012). In a study involving somatosensory stimulation in absence of movement, oscillations in the cerebellum measured with MEG were enhanced after the distortion of predicted somatosensory feedback (Tesche and Karhu, 2000). The cerebellar response was modulated as a function of expectancy and attention. Is this result a question of timing or sensory expectation? In his comment of this result, Richard Ivry considered the cerebellar response “to be best characterized as a detector of change or deviation in the sequence of sensory events, […] yet the cerebellar response is not strictly sensory in that it does not require the delivery of an actual stimulus” (Ivry, 2000). This suggests that the “expected” aspect of the presence or absence of sensory feedback is the key factor for a cerebellar anticipatory response (Chabrol et al., 2019). In this sense, the cerebellum has the functional and anatomical properties to predict and anticipate events.

### Sensory Attenuation

The sensory feedback that is generated by our voluntary actions elicits smaller cortical responses as compared to externally generates sensory signals. This phenomenon is known as “sensory attenuation” and is thought to rely on the forward model (Blakemore et al., 2000). As stipulated earlier, an efference copy of the motor command sent to the muscles would be used by the forward model to predict the sensory consequences of the command. The predicted consequences are compared to somatosensory feedback: if these match perfectly, cortical perceptual systems may not fully process the afferent signal, as it adds no information to the prediction (Haggard and Whitford, 2004). It has thus been proposed that sensory attenuation could be a process associated with the feeling of agency, as it allows us to distinguish self-produced as opposed to externally generated sensory stimuli (Blakemore et al., 1998, 2000).

The cerebellum could play a particular role in sensory attenuation, by evaluating the degree of matching between the predictions with the actual feedback. In agreement with this view, cerebellar activity is decreased in response to self-generated movements associated with tactile stimulation, while this activity is increased by external tactile stimulation (Blakemore et al., 1998). A recent study showed that disrupting cerebellar activity with TMS interfered with the cortical sensory attenuation of self-initiated sounds (Cao et al., 2017). In keeping with this, electrophysiological recordings of the cerebellum in non-human primates demonstrated that cerebellar neurons can cancel the reafferent sensory effects produced by self-generated movements (Brooks and Cullen, 2013).

### Cerebellum and Forward Model During Motor Control and Learning

In this part, we will present the pieces of evidence supporting the role of the cerebellum in different motor processes that rely on the forward model: on-line correction of movement, visuomotor adaptation, and conditional learning. Indeed, the ability to control our movement and to predict the movement outcome are two key processes for self-agency. Showing the involvement of the cerebellum in these processes would bring further arguments to explain its involvement in the sense of agency.

#### Online Motor Control and Rapid Corrections

There is an apparent contradiction between the rapidity of fast-tracking hand movements and the duration of sensory feedback processing. The latter appears to be too long to directly influence hand trajectory during fast movements (Desmurget and Grafton, 2000). In other words, because it takes time for sensory afferences to be processed, there is always a lag between the actual state of the motor effectors and how this state is perceived by the central nervous system. It has thus been proposed that on-line motor control relies on a forward model. The forward model integrates both the efference copy of the motor command and the sensory feedback to produce a prediction of the sensory consequences of the motor command. These predictions are directly compared with the sensory feedback generated by the movement and thus provide an optimal estimate of the state of the effector; any discrepancy would be used to correct the on-going movement (Miall et al., 1993; Wolpert et al., 1998; Desmurget and Grafton, 2000; Tanaka et al., 2020). On-line motor correction using the forward model would be faster than the processing time of using the sensory feedback alone. Empirical observations in healthy subjects of online motor corrections are consistent with the timing estimated with the forward model (Miall et al., 1993; Wolpert et al., 1998; Desmurget and Grafton, 2000). Patients with cerebellar lesions are impaired for on-line movement correction: overshooting or undershooting the target (dysmetria) and oscillatory corrections (intention tremor) are the hallmarks of cerebellar ataxia, which can be broadly defined as inaccuracy and incoordination in limb movements and instability in posture, gait and ocular saccades (Holmes, 1939; Cabaraux et al., 2020). Disrupting the lateral cerebellum with transcranial magnetic stimulation in healthy subjects alters on-line control of the ongoing movement (Miall et al., 2007). The cerebellum was thus proposed to act as a forward model during on-line motor control (Miall et al., 1993, 2007; Wolpert et al., 1998; Desmurget and Grafton, 2000). In particular, it was shown that cerebellar patients rely more importantly on visual feedback as compared to healthy controls as if they were missing an internal forward model (Day et al., 1998; Bhanpuri et al., 2013; Kakei et al., 2019; Tanaka et al., 2020; Zimmet et al., 2020). Reciprocally, an erroneous forward model recapitulates the tracking deficits observed in cerebellar ataxia (Miall et al., 1993). Altogether, this suggests that some of the deficits observed in cerebellar patients could be explained by an impaired forward model resulting in increased dependence on delayed visual feedback (Kakei et al., 2019; Cabaraux et al., 2020; Tanaka et al., 2020; Zimmet et al., 2020).

The role of the cerebellum in the forward model during rapid motor corrections seems to depend on the nature of sensory information that is relevant during movement execution. Proprioceptive feedback present two advantages compared to visual feedback: they are not easily deceived since they are directly related to the movement’s results in healthy individuals, and they are more rapidly processed by the central nervous system as compared to other sensory modalities. Optimal multisensory integration and feedback control in real-time rely more importantly on proprioceptive information (Crevecoeur et al., 2016). Once again, findings in pathophysiological models involving patients with cerebellar impairment allow narrowing this question. Cerebellar patients have an active proprioceptive deficit consistent with disrupted movement prediction rather than an inability to enhance peripheral proprioceptive signals during the action (Bhanpuri et al., 2013). Besides, cerebellar patients can also show a reduced feedback gain in situations where responses are driven by proprioception more than vision (Kurtzer et al., 2013). When the responses are driven by enhanced visual feedback, cerebellar patients rely on time-delayed cursor feedback of their hand position and appear unable to generate predictions of their hand position (Zimmet et al., 2020). Altogether, these results suggest that a fundamental property of the cerebellum in the forward model is the integration of proprioceptive information during the control of body movements.

### Visuomotor Adaptation

Visuomotor rotation tasks induce a discrepancy between the movement of the limb and the visual feedback. In such tasks, the repetition of trials results in a gradual reduction of the error, known as visuomotor adaptation. Visuomotor adaptation of reaching movement is a form of implicit motor learning that relies on the updating of a forward model through sensory prediction error (sPE). This forward model tends to minimize the discrepancy between the anticipated motor outcome and the actual sensory feedback to optimize the motor performance trials after trials (Mazzoni and Krakauer, 2006; Krakauer, 2009). Several lines of evidence suggest that the cerebellum is involved in updating the forward model during sensorimotor adaptation. First, patients with cerebellar lesions are impaired in visuomotor rotation tasks (Weiner et al., 1983; Martin et al., 1996; Smith and Shadmehr, 2005; Tseng et al., 2007; Rabe et al., 2009; Werner et al., 2010; Schlerf et al., 2013; Bernard and Seidler, 2013; Burciu et al., 2014). Second, neuroimaging studies repeatedly confirmed that cerebellar activity is increased during visuomotor adaptation in healthy subjects (Bernard and Seidler, 2013; Küper et al., 2014; Tzvi et al., 2020). Last, different modalities of cerebellar stimulation with transracial direct current stimulation (tDCS) in healthy volunteers can lead to improved or impaired visuomotor adaptation (Galea et al., 2011; Herzfeld et al., 2014; Yavari et al., 2016), strongly suggesting that the cerebellum is a key area for the forward model.

The cerebellum is not the only brain region required for on-line motor correction and motor learning, and the involvement of the striatum in this process has been reported (Graybiel, 2008; Doyon et al., 2009; Seidler et al., 2013). Interestingly, both cerebellar patients and patients with striatal degeneration (Huntington’s disease) are impaired at on-line movement correction. However, while Huntington’s patients were able to adapt to an external perturbation and improved their motor performance from trial to trial, cerebellar patients did not. This is consistent with the model of motor learning proposed by Doyon et al. (2009), which distinguishes motor sequence learning (incremental acquisition of a sequential movement) from motor adaptation (compensation for environmental changes). During the early encoding phase, motor sequence learning and motor adaptation recruit the same cerebral structures, involving the striatum and the cerebellum. The interaction between these two structures is thought to be critical for establishing new motor routines (Hoshi et al., 2005; Doyon et al., 2009). It is only later that these two types of learning are distributed over distinct cerebral structures: while motor adaptation relies more on the cerebellum, motor sequence learning and habit formation rather rely on the striatum (Graybiel, 2008; Doyon et al., 2009). Indeed, patients with basal ganglia disorders are not impaired during motor adaptation tasks (Seidler et al., 2013). The mechanisms underlying motor control and learning in the striatum and the cerebellum are thus different: while the cerebellum seems to provide a substrate for motor adaptation through the updating of a forward model, it is not the case for the striatum (Smith and Shadmehr, 2005; Graybiel, 2008; Seidler et al., 2013).

### Instrumental Conditional Learning

Instrumental conditional learning, also called operant conditioning, refers to the mechanism of creating the relationship between the stimulus and motor response to obtain a reward and to avoid punishment. Cerebellar Purkinje cells generate conditioned response and through the connections with the inferior olive, regulate the signal from the unconditional stimulus (Rasmussen and Hesslow, 2014). A growing body of evidence suggests that the cerebellum is also involved in reward processing and that the cerebellum learns to select the correct action *before motor execution*. Tracing and optogenetic activation of cerebellar projections in mice show that the cerebellum sends an excitatory efferent signal to the ventral tegmental area (VTA; Carta et al., 2019). The VTA is one of the regions sending brain-wide dopaminergic projections that represent the major pathways by which the brain controls reward and motivational behaviors. The existence of such a pathway would explain how repeated stimulation of the cerebellum increases dopamine in the mouse medial prefrontal cortex (Rogers et al., 2011). Second, the activity pattern of cerebellar cells is consistent with its active contribution during conditional learning tasks. Recent animal studies show that some cells located in sensorimotor areas of the cerebellar cortex modulate their activity in response to the reward, this modulation being stronger when the reward is unexpected (Heffley et al., 2018). Also, these cells fire in anticipation of the reward, when forelimb movements are correctly executed. This activity pattern resembles reward prediction error (rPE) signals recorded in the ventral striatum or the prefrontal cortex. Contrary to the striatal and prefrontal responses, cerebellar responses would be related to reward expectation, regardless of its valence (Kostadinov et al., 2019). Altogether, it seems that in addition to the error-related signal, the cerebellum is involved in selecting correct movements by processing reward-related signals to reinforce motor responses and to associate them with the dopaminergic release. Thus, the cerebellum can participate in motor selection by considering the probability of motor outcomes to be correct and rewarding, which would be important information to consider for the involvement of the cerebellum in the sense of agency.

## Discussion: Cerebellum and The Sense of Agency

Although the validity of the forward model has been questioned regarding the role of the cerebellum in cognitive processes (Sokolov et al., 2017; Diedrichsen et al., 2019), there is a growing body of evidence suggesting the role of the cerebellum in the sense of agency. Indeed, regardless of the forward model, the specific role of the cerebellum in the self-agency is poorly understood, but the cerebellum has been related to several aspects of actions goal-directness and self-attribution, which are the fundamental feature of intentional actions (Haggard, 2008). In line with our previous sections, we link motor control and the feeling of agency in a twofold manner. First, proper motor control is necessary for establishing a sense of agency. Second, these two processes might be supported by the same computation: the forward model. Here, we will also present some examples of movement disorders which could illustrate the role of the cerebellum in disrupted agency.

In the comparator model of agency, which is derived from the forward model of motor control (Synofzik et al., 2008b; Haggard, 2017), the implicit feeling of agency results from a match between the intentional content of the action and the actual sensory feedback generated by the movement. According to this model, a discrepancy in this comparison would result in a reduced or absent sense of agency (Haggard, 2017). A forward model is necessary for the feeling of agency, although it is not sufficient to explain the explicit judgment of agency (Synofzik et al., 2008b). The sense of agency thus seems to rely only partially on a forward model. Several arguments support the role of the cerebellum in the comparator model of agency. First, the cerebellum is a major region contributing to the sense of body ownership, described as “a feeling of mineness” that we experience toward our body parts (Tsakiris, 2010). Several empirical and experimental studies pointed to the strong interaction between the sense of body ownership and sense of agency, which usually mutually strengthened each other if they co-occur (Braun et al., 2018). It has thus been proposed that body ownership might rely on a forward model (Grechuta et al., 2019), and patients with cerebellar ataxias, a group of disorders characterized by cerebellar degeneration, showed an abnormally reduced sense of body ownership, evaluated by the rubber hand illusion experience (Fiorio et al., 2014). Second, as shown in the previous section, the cerebellum is involved in detecting a mismatch between the expected and actual sensory feedback, leading to a feeling of disrupted agency (Blakemore et al., 2001; Nahab et al., 2011; Seghezzi et al., 2019). For instance, in conversion tremor, patients exhibit involuntary postural tremor, leading to a mismatch between the intended movement and the actual movement results. In this case, aberrant motor symptoms critically use voluntary motor pathways, but patients experience the movements as involuntary, despite the absence of neurological causes for these symptoms. In conversion tremor patients, a task eliciting conversion tremor (posture specific) was compared to a task involving a voluntary mimic of the tremor (Voon et al., 2010). The authors found that a network involving the temporo-parietal junction and the cerebellum had decreased connectivity during conversion tremor. They suggested that this finding may reflect the lack of an appropriate sensory prediction signal, which would lead to the perception that the conversion movement is not self-generated.

In some of the experimental paradigms testing disrupted agency, the conditions artificially induced a mismatch by deceiving the participant with the manipulation of one modality of sensory feedback (introducing a delay between the movement and the production of an auditory tone or distorting the visual feedback for instance). As a result, brain activation during such tasks could be attributed to the realization of this deception and to a “simple mismatch” without agency disruption. This is especially true for healthy participants who do not have impaired motor control and who can rely on proprioceptive feedback. In other words, the increased cerebellar activity in these studies could be related to the detection of an inter-sensory mismatch between the proprioceptive feedback generated by the movement and the erroneous visual or auditory feedback. Alternatively, the increased cerebellar activity could be associated with the detection of a mismatch between the anticipation of the movement outcome and the actual sensory feedback, as postulated by the forward model. To disentangle these two possibilities, a recent study manipulated the visual feedback produced by hand movements during active and passive movements (van Kemenade et al., 2019). The authors introduced a temporal delay between the actual movement and the displayed image of that movement. They did so in two different conditions: in the active condition, the movement was initiated by the subject, whereas in the passive condition, the hand movement was generated by an external device. In both situations, the proprioceptive feedback generated by the hand movement was identical, but according to the forward model, the comparison between the efference copy and the sensory feedback generated by the movement should occur only in the active condition. Accordingly, the cerebellar activity was positively correlated to the delay between the movement and the visual feedback, specifically in the active condition, confirming its role as a *comparator* in the framework of the forward model. By contrast, other brain regions such as the temporo-parietal junction were sensitive to a mismatch in both active and passive conditions, suggesting a more general role in detecting inter-sensory mismatch (van Kemenade et al., 2019). These results strongly suggest that the cerebellum contributes directly to the implicit feeling of agency by comparing the anticipation of the movement outcome with the actual sensory feedback. This hypothesis is further supported by a recent study on the neural correlates of intentional binding (Zapparoli et al., 2020b). Compared to previous studies that explored the network underlying disrupted agency, this recent work used a more “physiological” approach by identifying the neural substrate of intentional binding, an implicit measure of self-agency (see “The Sense of Agency and the Forward Model” section). They showed that among other brain regions, the cerebellum activity positively correlated with the measure of intentional binding, providing strong evidence of its involvement in the feeling of agency (Zapparoli et al., 2020b).

As discussed in “The Sense of Agency and the Forward Model” section, the sense of agency is thought to rely on two distinct processes: the low-level, implicit, sensorimotor feeling of agency which relies on a forward model, and the higher-order, conceptual, and explicit judgment of agency (Synofzik et al., 2008b). We reviewed an accumulating body of evidence supporting the role of the cerebellum in the implicit feeling of agency: (i) the cerebellum presents the anatomical and functional properties required for a forward model; (ii) the cerebellum is involved in detecting a mismatch between the anticipated and actual sensory consequences of the movement; and (iii) activity in the cerebellum is correlated to the measure of the intentional binding, an implicit measure of the feeling of agency. The role of the cerebellum in the feeling of agency makes it necessary, but not sufficient, to establish the sense of agency. Indeed, although the feeling of agency and the judgment of agency both contribute to the sense of agency, they rely on distinct mechanisms. For instance, an explicit judgment of agency is possible despite a “non-agency” feeling (Synofzik et al., 2008b). Also, patients with lesions in the parietal lobe—which is a key region involved in detecting a mismatch between the intended movement and the movement’s results (Synofzik et al., 2008b; Haggard, 2017)—still have an agency judgment (Sirigu et al., 1999). By contrast, patients with prefrontal lesions can adapt to spatial sensorimotor discrepancies, yet they are unable to consciously detect these mismatches (Slachevsky et al., 2001). A recent example may illustrate the fact that the cerebellum is needed to properly detect mismatch, and that this mismatch information would then be used by associative areas to evaluate the explicit measure of the sense of agency. Delorme et al. (2016) used an explicit agency task in which participants had to catch targets with a cursor by moving a computer’s mouse. The control over the cursor could be disrupted by adding a spatial or a temporal discrepancy between the mouse and the cursor’s movements. The authors measured the level of the perceived discrepancies by the participants, who reflect them in metacognitive judgments of agency on an analogic scale. This task was performed by patients with cervical dystonia and healthy controls. Noteworthy, cerebellar dysfunction has an important if not a major contribution to dystonia (Neychev et al., 2008), including cervical dystonia (Popa et al., 2018). Dystonic patients explicitly reported being more in control in the temporal discrepancy condition than healthy participants, suggesting that they failed to detect any mismatch between their intended movements and the perceived feedback of the timing of their action. The implication of the cerebellum could only be hypothesized, because of the absence of neurophysiological data that could be associated with the observed behavior. Patients with Gilles de la Tourette’s syndrome (GTS), a hyperkinetic movement disorder with tics, also showed a “disturbed” agency in explicit and implicit agency tasks (Delorme et al., 2016; Zapparoli et al., 2020a). Specifically, in this patient population, weaker level of intentional binding was associated with disease severity as well as a silencing of the cerebello-parieto-premotor network (Zapparoli et al., 2020a) usually related to intentional binding (Zapparoli et al., 2020b). This suggests that the cerebellum is an element of the agency network and that this network can be affected by the unreliability of the motor output due to involuntary movements present in various movement disorders. In some movement disorders, voluntary movements are always accompanied by an involuntary one. In severe forms of essential tremor involving cerebellar pathways, patients might not feel in full control of their actions during voluntary movements due to systematic action tremors. If the involuntary movement is systematic, patients can anticipate that their movement is going to be disrupted. To our knowledge, implicit or explicit measures of the agency were never investigated in pathologies in which involuntary and voluntary movements co-occur.

We hypothesize that the cerebellum, together with the posterior parietal cortex, is part of a neural network that is involved in comparing the predicted movement outcome (through the integration of the efference copy) with the actual sensory feedback generated by the movement. This first step would be involved in the implicit feeling of agency, and the result of this comparison would then be transmitted to higher-order associative areas in the prefrontal cortex, where it would be integrated along with contextual knowledge and belief reasoning to give rise to the judgment of agency (Synofzik et al., 2008b). At the level of the cerebellar module, the deep cerebellar nuclei may integrate both the efference copy and the sensory feedback through the inputs of the Purkinje cells and the mossy fibers, as is the case for motor control (Tanaka et al., 2020). The sensory prediction error would then be transmitted to different cortical regions through parallel loops: to the parietal cortex to establish the feeling of agency, and to the motor regions to serve motor control (Figure 1).

## Conclusion

The sense of agency depends upon a set of mechanisms involving the processing of specific neural signals, from sensory as well as from central origin. The first one, the implicit feeling of agency, would relate to the action monitoring and predictive processes; the second one, the explicit judgment of the agency, would relate to the high-order mechanism. The first level provides an immediate signal for controlling and adapting actions to their goal during action execution and is thought to rely on the principle of congruence of the action-related signals through the forward model. In contrast, the second level provides information about the intentions, plans, and desires of the author of these actions. These two levels are interdependent and together contribute to elaborate the sense of agency along the action execution. Here, we brought a conceptual analysis of empirical data that lead us to consider the role of the cerebellum in the implicit feeling of agency. In support of this view, Table 1 illustrates the functional involvement of parts of the cerebellum in the references cited in the different sections. As such, the cerebellum is necessary, but not sufficient, to establish a sense of agency. Indeed, direct proof of the involvement of the cerebellum in the explicit judgment of agency is still missing and we could only infer some working hypotheses that need a demonstration.

**Table 1:** Cerebellar functional anatomy related to functions associated with the forward model and the sense of agency.

Function	Article	Experimental design	Cerebellar region involved
	Diedrichsen et al. (2005)	fMRI correlate of target error (unpredictable change in target location)	Lobules V, VI, VIII and dentate nucleus
Detection of sensory prediction errors		fMRI correlate of target error (unpredictable change in target location) fMRI correlate of execution error (alteration of visual feedback)	Lobules V, VI, VIII and dentate nucleus
	Schlerf et al. (2012)	Error detection	Lobules V and VI
	Blakemore et al. (2001)	Correlation of cerebellar activity with the abnormal delay of sensory feedbacks	Border of lobule VI and crus II
	van Kemenade et al. (2019)	Correlation of cerebellar activity with the abnormal delay of sensory feedbacks	Lobule V
	Nahab et al. (2011)	Correlation with the loss of control	Left cerebellar tonsil, left cerebellar pyramid
On-line motor control	Miall et al. (2007)	Cerebellar stimulation with TMS impairs on-line motor control	Lateral cerebellum
Sensory attenuation	Blakemore et al. (1998)	Decreased cerebellar activation in response to self-generated tactile stimulus	Right anterior cerebellar cortex
	Brooks and Cullen (2013)	Electrophysiological recordings in the cerebellum of non-human primates suggest a role in the cancellation of self-produced afferences
	Cao et al. (2017)	Cerebellar stimulation with TMS alters the cortical sensory attenuation of self-generated sounds	Lateral cerebellum
Visuomotor adaptation	Bernard and Seidler (2013)	A Meta-analysis of fMRI and PET study exploring visuomotor adaptation	Lobule IV
	Küper et al. (2014)	fMRI study of visuomotor adaptation	Lobule VIII and caudal dentate nucleus
	Tzvi et al. (2020)	fMRI study of visuomotor adaptation	Lobule VIII, crus II, lobule VI, crus I
	Galea et al. (2011)	tDCS over the cerebellum causes faster adaptation during visuomotor adaptation	Right cerebellar cortex
	Yavari et al. (2016)	tDCS over the cerebellum alters localization of the hand after a movement without visual feedback	Right cerebellar cortex
Conditional learning	Carta et al. (2019)	In mice, cerebellar nuclei send projections to the VTA and modulate the reward pathway	Deep cerebellar nuclei
	Rogers et al. (2011)	In mice, stimulation of the cerebellar nuclei triggers. Dopamine release in the medial prefrontal cortex	Dentate nucleus
	Heffley et al. (2018)	In mice, climbing fibers responses in the lateral cerebellum encode reward prediction	Lateral cerebellum
	Kostadinov et al. (2019)	In mice, the cerebellum encodes reward prediction	Lobule simplex
Anticipation	Tesche and Karhu (2000)	MEG study exploring the event-related potential during sensory ommission	Lateral cerebellum + vermis
	Cui et al. (2000)	Event-related during a delayed sequential finger movement task	Cerebellum lobules VI
Sense of agency	Seghezzi et al. (2019)	A Meta-analysis of fMRI study exploring the sense of agency	Right cerebellum lobule VI
	Zapparoli et al. (2020b)	fMRI study of the cerebral regions which activity correlates with the intentional binding	Cerebellum lobules IV and V

*Anatomical specifications of the different cerebellar structures involved in the references listed in the manuscript*.

We consider that the role of the cerebellum in the feeling of agency is twofold: first, the cerebellum ensures the quality control of movements, which is a necessary condition for the establishment of the sense of agency; second, recent evidence suggests that the cerebellum is directly involved in the sense of agency by comparing the intentional content of our actions with their outcomes, a process that is thought to rely on a forward model.

## Author Contributions

QW, YW, and CG conceived of and wrote the article. All authors contributed to the article and approved the submitted version.

## Conflict of Interest

The authors declare that the research was conducted in the absence of any commercial or financial relationships that could be construed as a potential conflict of interest.
